# Genotypic Diversity of *Staphylococcus aureus* α-Hemolysin Gene (*hla*) and Its Association with Clonal Background: Implications for Vaccine Development

**DOI:** 10.1371/journal.pone.0149112

**Published:** 2016-02-11

**Authors:** Meng Xiao, Rui Zhao, Qi Zhang, Xin Fan, Matthew V. N. O’Sullivan, Dong-Fang Li, Xin-Ying Wang, Hong-Long Wu, Fanrong Kong, Ying-Chun Xu

**Affiliations:** 1 Department of Clinical Laboratory, Peking Union Medical College Hospital, Chinese Academy of Medical Sciences, Beijing, China; 2 Department of Laboratory, Beijing Electric Power Hospital, Beijing, China; 3 Centre for Infectious Diseases and Microbiology Laboratory Services, ICPMR – Pathology West, Westmead Hospital, University of Sydney, Westmead, New South Wales, Australia; 4 Marie Bashir Institute for Infectious Diseases and Biosecurity, University of Sydney, Sydney, New South Wales, Australia; 5 Binhai Genomics Institute, BGI-Tianjin, BGI-shenzhen, Tianjin, China; 6 Tianjin Translational Genomics Center, BGI-Tianjin, BGI-shenzhen, Tianjin, China; University of Mississippi Medical Center, UNITED STATES

## Abstract

The α-hemolysin, encoded by the *hla* gene, is a major virulence factor in *S*. *aureus* infections. Changes in key amino acid residues of α-hemolysin can result in reduction, or even loss, of toxicity. The aim of this study was to investigate the diversity of the *hla* gene sequence and the relationship of *hla* variants to the clonal background of *S*. *aureus* isolates. A total of 47 clinical isolates from China were used in this study, supplemented with *in silico* analysis of 318 well-characterized whole genome sequences from globally distributed isolates. A total of 28 *hla* genotypes were found, including three unique to isolates from China, 20 found only in the global genomes and five found in both. The *hla* genotype generally correlated with the clonal background, particularly the multilocus sequence type, but was not related to geographic origin, host source or methicillin-resistance phenotype. In addition, the *hla* gene showed greater diversity than the seven loci utilized in the MLST scheme for *S*. *aureus*. Our investigation has provided genetic data which may be useful for future studies of toxicity, immunogenicity and vaccine development.

## Introduction

*Staphylococcus aureus* is one of the leading human pathogens worldwide. It causes a broad range of diseases from superficial infections to life-threatening invasive diseases. Antimicrobial therapy is sometimes ineffective, owing to the development of antimicrobial-resistant strains, such as methicillin-resistant *S*. *aureus* (MRSA) [1]. *S*. *aureus* expresses various virulence factors, including a broad range of exotoxins. Some of these toxins, such as most of the phenol-soluble modulins and α-hemolysin (also known as α-toxin), are encoded in the core-genome; while others, such as Panton-Valentine leucocidin, are encoded by acquired mobile genetic elements [2, 3].

The α-hemolysin, which belongs to a class of small β-barrel pore-forming cytotoxins, is a major virulence factor in *S*. *aureus* infections [2, 4]. It is encoded by the 960-bp *hla* gene, which is initially produced as a 319-residue precursor, then processed to a 293-residue (approximately 33 kDa) mature toxin [4, 5]. Previous studies have proven that changes in key amino acid residues of α-hemolysin, such as a histidine substitution at amino acid 35, can result in reduction or even loss of virulence [6, 7].

In this study, we determined the genotypes of *hla* in 47 *S*. *aureus* isolates collected in China, and compared this with the clonal background of the isolates. For comparison, *hla* genotype and clonal background were also determined for well-characterized and published whole genome sequences of 318 global strains by *in silico* analysis.

## Methods

### Ethics

The study was approved by the Human Research Ethics Committee of Peking Union Medical College Hospital (No. S-263). Written consent was obtained from patients, and the study was carried out in accordance with approved guidelines.

### *Staphylococcus aureus* isolates

Isolates were collected from patients with *S*. *aureus* infections in Beijing Electric Power Hospital during January 2011 to June 2011. A total of 47 *S*. *aureus* isolates were collected consecutively, each from different individuals. The most common type of infection was pneumonia (n = 31, 66.0%), followed by soft tissue infection (n = 6, 12.8%), urinary tract infection (n = 6, 12.8%), bloodstream infection (n = 2, 4.3%), joint infection (n = 1, 2.1%), and gallbladder infection (n = 1, 2.1%).

### Molecular identification of isolates and *mecA* gene detection

DNA extraction of *S*. *aureus* isolates was performed as previously described [8]. A multiplex PCR was used for simultaneous amplification of 16S rRNA, *femA* and *mecA* genes for identification and differentiation of methicillin-susceptible *S*. *aureus* (MSSA) and MRSA isolates [9].

### Genotyping of the 960-bp *hla* gene

Primer pair of hlaF1 (5’- TTAGCCGAAAAACATCATTTC-3’) and hlaR2 (5’- TTATTCCCGACGAAATTCCAA-3’) was designed for amplification of the complete 960-bp *hla* gene encoding the α-hemolysin precursor, which was made up of a 78-bp nucleotide sequence encoding the 26 aa signal peptide, followed by the 882-bp nucleotide sequence encoding the 293 aa mature α-hemolysin.

Each PCR mix contained 12.5 μL of 2× EasyTaq PCR SuperMix (TransGen Biotech, Beijing, China), 2 μL of DNA template, 0.5 μM of each forward and reverse primer, and molecular biology grade water (TransGen Biotech) added to make a total volume of 25 μL. PCR was performed as follows: initial denaturation at 95°C for 15 min, followed by 30 cycles of 94°C for 2 min, 55°C for 2 min, 72°C for 2 min, with a final extension at 72°C for 10 min. The products were sequenced in both directions using the inner primer pair hlaF2 (5’- GAAGTTATCGGCTAAAGTTATAA-3’) and hlaR1 (5’- CATAATTAATACCCTTTTTCTC-3’) on the DNA analyzer ABI 3730XL system (Applied Biosystems, Foster City, CA).

The obtained 960-bp *hla* gene sequences were compared to a wild-type reference sequence from *S*. *aureus* strain WOOD 46 (GenBank accession no. X01645) [10], aligned using CLC sequence viewer (version 7, QIAGEN Aarhus, Denmark) to detect single nucleotide polymerases (SNPs), and designate genotypes (the *hla* DNA sequence was identical for all isolates belonging to a given genotype). Further, the α-hemolysin peptide sequences of isolates were deduced and aligned to determine the presence of amino acid substitutions.

### Assignment of clonal background

All isolates were analyzed by multilocus sequence typing (MLST) and *spa* typing using previously established methods [11, 12]. Assignment of related sequence types (STs) into clonal complexes (CCs) was conducted using eBURST [13]. In addition, all MRSA isolates were characterized by staphylococcal cassette chromosome *mec* (SCC*mec*) typing as described by Chen et al. [14]. *S*. *aureus* clones were named in the format of ST-*spa* type or ST-SCC*mec* type-*spa* type (e.g. ST5-t002 for a MSSA clone, or ST239-III-t030 for a MRSA clone).

### *In silico* analysis of published whole genome sequences

Because only limited number of *S*. *aureus* isolates was involved in the present study, to better define the *hla* gene diversity and association with clonal background amongst *S*. *aureus* with more different genetic background, 318 selected well-characterized published genomes derived from other geographic regions were further studied. The 318 selected published genomes included i) genetic information of all *S*. *aureus* isolates with complete assembled whole genome sequences as at July 8 2015 were obtained from the NCBI Genome database (70 isolates, S1 Table), and ii) *S*. *aureus* genome sequences from four previous publications (248 isolates, S2 Table) [15–18]. These genomes comprised examples from the major worldwide lineages of MRSA, e.g. CC8 (including USA-300), CC1 (including USA 400), CC22 (including EMRSA15), CC93 (including Queensland CA-MRSA), CC30 (including EMRSA16) and CC121 isolates.

The MLST STs and *hla* genotypes of the above isolates were determined by *in silico* mapping the paired-end reads of 318 isolates to seven gene loci sequences of *S*. *aureus* ST1 and wild-type *hla* reference sequence (GenBank accession no. X01645), respectively, using Burrows-Wheeler Alignment [19]. Base coverage of each position of genes was assessed using SAMtools mpileup packages (http://samtools.sourceforge.net/mpileup.shtml). The support number of reference base (ref) and alternative base (alt) are examined at each position in each strain. High quality SNPs were defined when SNPs satisfied the criteria of alt/(alt+ref)>0.8. According to these SNPs, the ST and *hla* genotype for each isolate was identified.

MLST STs and *hla* genotypes were entered into BioNumerics software v7.5 (Applied Maths, Austin, TX) for minimum-spanning-tree analysis. The diversity of the sequences of the *hla* gene and seven genes utilized in the MLST scheme for *S*. *aureus* was analyzed by DnaSP (version 5.1, University of Barcelona, Spain).

## Results

### Molecular identification and detection of the *mecA* gene

All 47 isolates were confirmed as *S*. *aureus* by molecular methods. Thirty-three isolates (70.2%) were determined to be MRSA by detection of the *mecA* gene.

### Genotypes of the 960-bp *hla* gene

Amongst the 47 isolates from China, a total of eight *hla* genotypes (genotype 1 to 8) and six peptide sequence types were identified (Tables 1 and 2). Of the eight *hla* genotypes, genotype 1 was predominant (n = 27, 57% of 47 isolates), followed by genotype 3 (n = 7, 15%) and genotype 5 (n = 3, 6%). The remaining four genotypes were rare, with one or two isolates belonging to each (Table 1).

**Table 1:** Nucleotide mutations and corresponding amino acid substitutions of the eight *S*. *aureus hla* genotypes identified from 47 isolates from China.

	Nucleotide mutation position/corresponding amino acid substitution^a^																	
Genotype	-68	-22	-6&-5	144	177	399	438	453	479	606	624	669	708	765	777	824	864	GenBank accession no.
Wild type^a^	G	G	G G	C	T	C	G	T	C	T	T	G	A	T	T	C	A	X01645.1
Genotype 1	A/	A/	AA/	T/	—	—	—	—	—	C/	—	—	—	C/	C/	T/	—	KT279554.1
	R(-23)H	Syn	G(-2)N	Syn						Syn				Syn	Syn	T275I		
Genotype 2	A/	A/	AA/	T/	—	—	—	—	—	C/	—	—	G/	C/	C/	T/	—	KT279555.1
	R(-23)H	Syn	G(-2)N	Syn						Syn			Syn	Syn	Syn	T275I		
Genotype 3	—	A/	AA/	—	—	—	—	—	—	—	G/	—	—	C/	—	—	—	KT279558.1
		Syn	G(-2)N								D208E			Syn				
Genotype 4	—	A/	AA/	—	A/	T/	C/	G/	—	C/	—	T/	—	C/	—	—	—	KT279556.1
		Syn	G(-2)N		Syn	Syn	Syn	Syn		Syn		Syn		Syn				
Genotype 5	—	A/	AA/	—	—	—	—	—	—	—	—	—	—	—	—	—	—	KT279559.1
		Syn	G(-2)N															
Genotype 6	—	A/	AA/	—	—	—	—	—	—	—	G/	—	—	C/	—	—	T/	KT279557.1
		Syn	G(-2)N								D208E			Syn			Syn	
Genotype 7	—	A/	AA/	T/	—	—	—	—	—	C/	—	—	—	C/	C/	T/	—	KT279561.1
		Syn	G(-2)N	Syn						Syn				Syn	Syn	T275I		
Genotype 8	—	A/	AA/	T/	—	—	—	—	Del^b^	C/	—^b^	—^b^	—^b^	C/	C/	T/	—^b^	KT279560.1
		Syn	G(-2)N	Syn						Syn^b^				Syn^b^	Syn^b^	T275I^b^		

Abbreviations: Syn, synonymous mutation; Del, deletion mutation; N/A, not applicable; “—”, no mutation.

^a^Nucleotide/peptide positions were designated relative to the first nucleotide/amino acid of the mature α-hemolysin (*S*. *aureus* strain WOOD 46, nucleotide sequence GenBank accession no. X01645).

^b^The deletion at nucleotide position 479 of genotype 8 resulted in peptide termination at residue position 164.

**Table 2:** Relationship between *hla* genotypes and molecular clone backgrounds of 47 *S*. *aureus* isolates from China.

*hla* genotype	Molecular clone	Clonal Complex (CC)	No. of isolates
**Peptide sequence type 1**			
Genotype 1	ST239-MRSA-III-t030	CC8	13
Genotype 1	ST239-MRSA-III-t037	CC8	8
Genotype 1	ST239-MRSA-III-t2270	CC8	5
Genotype 1	ST239-MRSA-III-t459	CC8	1
Genotype 2	ST239-MRSA-III-t030	CC8	2
**Peptide sequence type 2**			
Genotype 3	ST5-MRSA-II-t002	CC5	3
Genotype 3	ST5-MSSA-t002	CC5	3
Genotype 3	ST25-MSSA-t078	CC25	1
**Peptide sequence type 3**			
Genotype 4	ST59-MRSA-IV-t437	CC59	1
Genotype 4	ST59-MSSA-t163	CC59	1
Genotype 5	ST1-MSSA-t127	CC1	3
**Peptide sequence type 4**			
Genotype 6	ST6-MSSA-t2467	CC6	1
Genotype 6	ST6-MSSA-t701	CC6	1
Genotype 6	ST7-MSSA-t091	CC7	1
Genotype 6	ST7-MSSA-t796	CC7	1
**Peptide sequence type 5**			
Genotype 7	ST188-MSSA-t189	CC1	1
**Peptide sequence type 6**			
Genotype 8	ST188-MSSA-t189	CC1	1

Eighteen SNPs were found, including four on the signal peptide encoding portion (nucleotide positions -68, -22, -6 and -5 relative to the first nucleotide of mature α-hemolysin portion), and 14 on the mature α-hemolysin encoding sequence (nucleotide positions 144, 177, 399, 438, 453, 479, 606, 624, 669, 708, 765, 777, 824 and 864) (Table 1). The majority of the SNPs (12/18, 67%) were synonymous—only five (28%, nucleotide positions -68, -6, -5, 624 and 824) were nonsynonymous. Lastly, a deletion mutation was detected in nucleotide position 479 of genotype 8, presumably resulting in a prematurely terminating transcript (Table 1). Of the 18 SNPs identified, nucleotide position -68 was ST239-specific, positions 177, 399, 438,453 and 669 were ST59-specific, position 765 was ST1-specific, whilst the other 11 were not lineage specific.

### Clonal background of *S*. *aureus* isolates

A total of six ST-SCC*mec*-*spa* types and nine ST-*spa* types were identified amongst 32 MRSA and 15 MSSA isolates from China, respectively (Table 2). 88% (29/33) of the MRSA isolates belonged to CC8, 9% (3/33) belonged to CC5, and 3% (1/33) belonged to CC59. The ST239-III-t030 clone comprised over half of the CC8 MRSA isolates (15/29, 52%), followed by ST239-III-t037 (8/29, 28%), ST239-III-t2270 (5/29, 17%) and ST239-MRSA-III-t459 (1/29, 4%). All three CC5 MRSA isolates were ST5-II-t002 (Table 2).

In comparison, the distribution of MSSA clones was more diverse, with no clone comprised of more than three isolates. ST5-t002 was represented by three MRSA and three MSSA isolates in this study, and ST59 was represented by one MRSA and one MSSA isolates with differing *spa* types. No other ST or CC was common to both MRSA and MSSA isolates (Table 2).

### Relationship between *hla* genotypes and clonal background

A strong correlation was observed between both *hla* genotypes and α-hemolysin peptide sequence types, and the clonal background of isolates from China.

Of the eight *hla* genotypes, six were restricted to either MRSA (genotypes 1 and 2) or MSSA (genotypes 5, 6, 7 and 8) strains. The predominant MRSA clone, ST239-MRSA-III-t030, was represented by *hla* genotype 1 (13/15 isolates, 86.7%) or genotype 2 (2/15 isolates, 13.3%). All of the remaining ST239-MRSA-III isolates possessed *hla* genotype 1. Both genotype 1 and 2 *hla* genes encoded α-hemolysin of peptide sequence type 1 (Table 2). *hla* genotypes 5 and 6 were found in one and four MSSA clones, respectively. Of the two ST188-MSSA-t189 isolates, one possessed *hla* genotype 7 and the other genotype 8, which differed by one deletion mutation at nucleotide position 479 (Table 1).

The remaining two *hla* genotypes, genotypes 2 and 3, were represented by both MRSA and MSSA isolates. Genotype 2 was found in six isolates of ST5-t002 (including three isolates each of ST5-MRSA-II-t002 and ST5-MSSA-t002) and one isolate of ST25-MSSA-t078. Genotype 3 was found in two ST59 isolates (one isolate each of ST59-MRSA-IV-t437 and ST59-MSSA-t163), and one isolate of ST1-MSSA-t127.

### The *in silico* analysis of 318 well-characterized genomes

Amongst 318 well-characterized *S*. *aureus* genomes, 25 *hla* genotypes were identified, including 20 genotypes not found in the 47 isolates from China (S1 and S2 Tables), for a total of 28 *hla* genotypes identified in this study. The SNPs identified for all clinical isolates and published genomes are summarized in S3 Table.

Substantial diversity amongst *S*. *aureus hla* gene was found. Compared to the seven loci utilized in the MLST, the *hla* gene had higher nucleotide diversity (0.0256 vs. 0.0042–0.0119), more haplotypes identified (28 vs. 10–19), greater haplotype diversity (0.899 vs. 0.517–0.804) and higher non-synonymous polymorphisms/ synonymous sites ratio (3.598 vs. 3.059–3.569) (Table 3). Of note, 79 of 107 ST22 *S*. *aureus* isolates and one ST188 isolate (*hla* genotype 8) had one to 16 deletion mutations in their *hla* sequences (S3 Table). In addition, all 20 ST36 isolates had a SNP C→T at sequence position 259, which resulted in a premature stop codon (S3 Table). These mutations would presumably inhibit production of the toxin protein.

**Table 3:** Comparison of genetic diversity between the *hla* gene and seven gene loci utilized in the multilocus sequence typing scheme.

		Multilocus sequence typing loci						
Characters	*hla*	*arcC*	*aroE*	*glpF*	*gmk*	*pta*	*tpi*	*yqiL*
Nucleotide diversity	0.0256	0.0083	0.0093	0.0042	0.0083	0.0079	0.0119	0.0073
No. of haplotypes	28	13	15	12	10	18	19	19
Haplotype diversity	0.899	0.760	0.796	0.517	0.726	0.804	0.800	0.799
No. of non-synonymous sites	730.09	350.45	356.18	350.45	327.30	362.89	312.13	400.19
No. of synonymous sites	202.91	105.55	99.82	114.55	91.70	111.11	89.87	115.81
No. of non-synonymous sites/ no. of synonymous sites	3.598	3.320	3.568	3.059	3.569	3.266	3.473	3.455574

**Fig 1:**
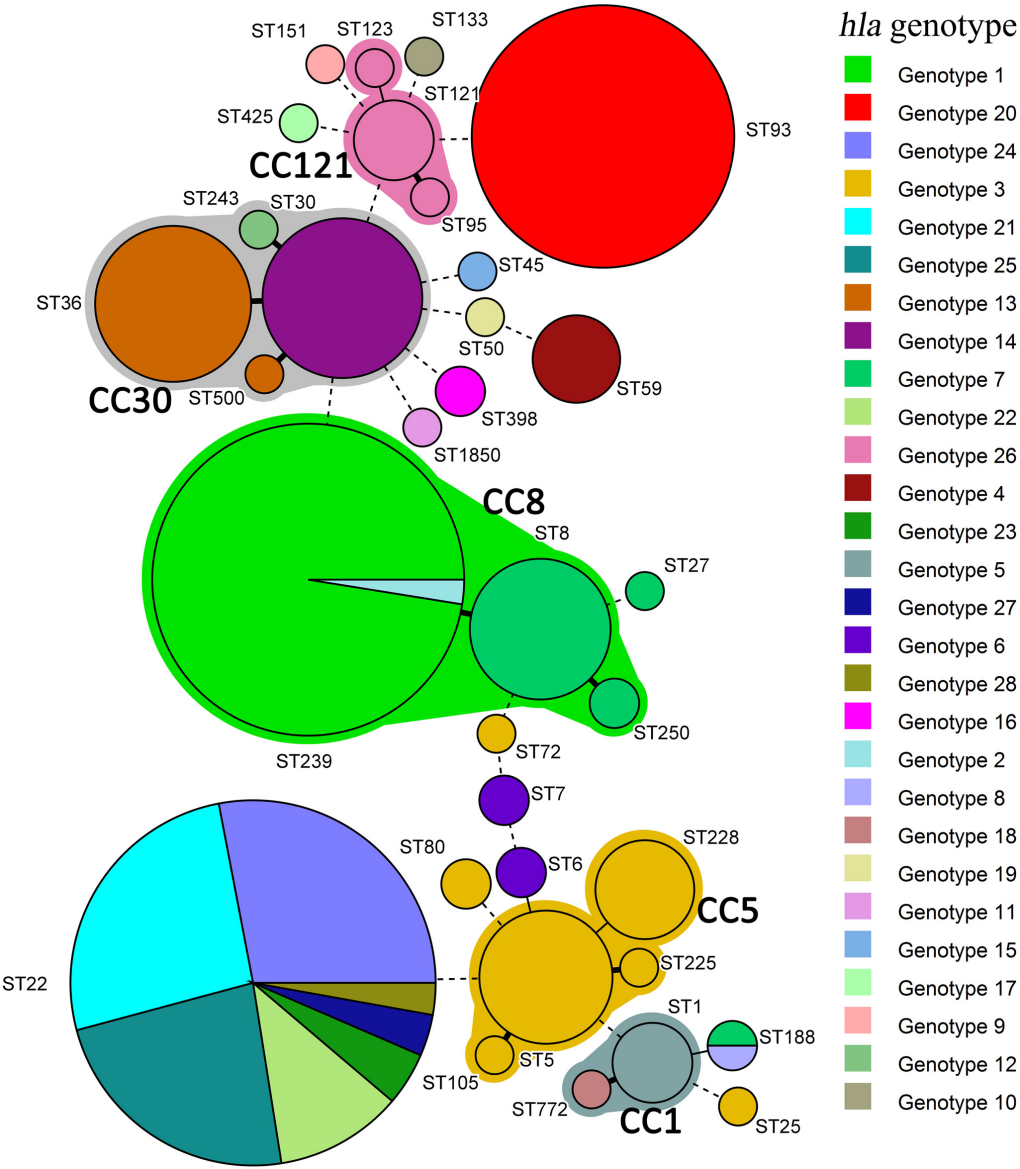
Minimum-spanning tree of MLST data from 47 *S*. *aureus* clinical isolates collected in China and 318 whole genome sequences. Circle sizes represent the number of isolates; circles are color coded by *hla* genotypes and labeled with the ST. Each circle represents a unique ST. Major clonal complexes (CCs), ie. CC8, CC5, CC30, CC121 and CC1, were labeled by green, brown, gray, pink and celadon colors, respectively. Thick solid lines, single-locus variant; Thin solid lines, double-locus variant; dashed lines, ≥3-locus variant.

In combining the 47 isolates from China and the 318 globally distributed genomes for composite analysis, it was again noted that *hla* genotype was closely related to the clonal background of the isolate, in particular the ST, with little association with the geographic origin, host source or methicillin-resistance phenotype (Table 2, S1 and S2 Tables and Fig 1). The minimum-spanning tree analysis of MLST data shown that for 30 of 33 STs identified in the present study, isolates belonging to the same ST shared a unique *hla* genotype, and ST22 was the only sequence type that comprised more than three (seven in all) *hla* genotypes (Fig 1). In addition, STs belonging to the same CC frequently shared the same or close-related *hla* genotype, e.g. ST5, ST105, ST225 and ST228 of CC5 was comprised of 24 isolates belonging to *hla* genotype 3, ST8 and ST250 of CC8 was comprised of 18 isolates belonging to *hla* genotype 7, and ST95, ST121 and ST123 of CC121 was comprised of seven isolates belonging to *hla* genotype 26 (Fig 1).

Although the same *hla* genotype may be shared by unrelated STs, this was observed uncommonly. For instance, one isolate each that belonged to ST72 and ST25 isolates were *hla* genotype 3, which was mostly associated with CC5 *S*. *aureus* isolates. Likewise, one ST188 isolate was identified as *hla* genotype 25, which was mostly associated with non-ST239 CC8 isolates.

## Discussion

Infections due to antimicrobial-resistant pathogens are a growing problem all over the world. In developing countries like India and China, antimicrobial resistance is particularly prevalent, owing to previous unregulated overuse of antimicrobials [20, 21]. *S*. *aureus* is one of the commonest Gram-positive bacterial pathogens, and in many places, the majority of *S*. *aureus* infections are now caused by multidrug-resistant strains, including MRSA and vancomycin-resistant *S*. *aureus* (VRSA). Immunotherapies are now being investigated as an alternative therapeutic options for staphylococcal infections in the hope that these may avoid the selection pressure associated with the use of antimicrobials [3, 22].

The *S*. *aureus* α-hemolysin was the first described of a family of bacterial pore-forming β-barrel toxins, which play an important role in the pathogenesis of staphylococcal disease [4, 23]. As such, it was chosen as a potential target for the development of vaccines to combat *S*. *aureus* infections, and positive results have been obtained in some preclinical trials targeting pneumonia and skin and soft tissue infections [23–26]. It has been noted that substitutions in amino acid residues may reduce the activity of α-hemolysin. For instance, a α-hemolysin mutant with a H35L substitution was found to have no hemolytic or lethal activity, despite retaining the ability to bind to target cells [6, 7]. The EMRSA-16 CC30 *S*. *aureus* isolates were another example. As observed in the present study, and as reported elsewhere, CC30-ST36 isolates had a SNP C→T at nucleotide sequence position 259, which resulted in a premature stop codon [27, 28]. It has been proven that CC30 isolates possessed this SNP had significantly reduced toxin production and decreased lethality in a mouse model [27]. These α-hemolysin mutants could be considered as candidate immunogens in prototypic *S*. *aureus* vaccines [23–26, 29].

Despite this work, little has been described regarding the genetic polymorphism of the *hla* gene in *S*. *aureus*. This is an important consideration, since variation in α-hemolysin peptide sequences could potentially lead to failure in antigen-antibody binding and thus compromise vaccine efficacy. In this study, we have illustrated the diversity of the *hla* gene in *S*. *aureus* and the relationship of *hla* sequence with clonal background, using 47 *S*. *aureus* clinical isolates from China supplemented with 318 well-characterized and globally distributed isolates with published whole genome sequences.

All ST239 from China were MRSA, and carried either genotype 1 or genotype 2 *hla*. These two genotypes differed by just one synonymous nucleotide mutation, (peptide sequence type 1). Amongst the 70 global *S*. *aureus* isolates with published genomes, seven isolates were ST239-MRSA-III, all of which also carried genotype 1 *hla*, regardless of the isolates’ geographic origins. The ST239-MRSA-III clone has been reported largely to be hospital-acquired and widely disseminated in Brazil, Australia, New Zealand and many Asian countries in the past decade, although the prevalence different *spa* types within this clone (e.g. *spa* type t030 and t037) vary in different regions [8, 30, 31]. In a previous genome-based phylogeographic analysis, it was determined that human movement played an important role in the global dissemination of ST239-MRSA-III [32]. Therefore, the consistent *hla* genotype of this clone across different regions is not surprising.

Interestingly, all of the 14 ST5 *S*. *aureus* isolates (six clinical isolates from China and eight global strains), including nine ST5-MRSA-II and five ST5-MSSA strains, possessed genotype 3 *hla*. Genotype 3 *hla* was also found in other CC5 *S*. *aureus* clones, including ST228-MRSA-I-t041 (n = 8), ST105-MRSA-II-t002 (n = 1) and ST225-MRSA-II-t003 (n = 1). Likewise all six ST59 isolates analyzed in this study, despite diverse methicillin-resistance phenotypes, SCC*mec* and *spa* types, carried genotype 4 *hla*. The ST59 lineage is primarily a community-acquired MRSA clone predominant in China and several other Asian countries [33]. These results again indicate that the *hla* genotype correlated closely with the ST.

Meanwhile, ST22 isolates shown significantly higher *hla* genotype diversity (comprised seven *hla* genotypes in all) than other *S*. *aureus* clones. Only occasional discrepancies between *hla* genotype and ST were observed. Future vaccine development will need to account for the influence of this diversity on vaccine effect.

## Conclusion

We have found substantial diversity amongst *S*. *aureus hla* gene and amino acid sequences. Strong correlations between *hla* genotypes and clonal background were found in *S*. *aureus*, regardless of the isolates’ geographic origins and methicillin-resistance phenotype. Although the relative virulence of different *hla* genotypes remain undetermined, our investigation has provided some preliminary epidemiologic data which will be essential for future vaccine development.

## Supporting Information

S1 Table *In silico* analysis of *hla* genotype and clonal background of 70 complete assembled whole genome sequences of *S*. *aureus*. (DOCX)

S2 Table *In silico* analysis of *hla* genotype and clonal background of and 248 *S*. *aureus* genomes from four previous publications. (DOCX)

S3 Table Alignment of 365 *hla* sequences obtained from 47 China clinical isolates and 318 published genomes, and SNPs identified. (XLSX)
