# Supplementary material for: Genotypic Diversity of Staphylococcus aureus α-Hemolysin Gene (hla) and Its Association with Clonal Background: Implications for Vaccine Development
Source: PLoS One. 2016 Feb 11;11(2):e0149112. doi: 10.1371/journal.pone.0149112 (PMC4750931; doi:10.1371/journal.pone.0149112)
Supplement: S1 Table — (DOCX) [file pone.0149112.s001.docx]

**S1 Table. *In silico* analysis of *hla* genotype and clonal background of 70 complete assembled whole genome sequences of *S. aureus.***

| **Strain** | ***hla* genotype** | **Clonal**  **complex (CC)** | **Sequence**  **type (ST)** | **Phenotype** | **SCC*mec***  **type** | ***spa***  **type** | **Country** | **Host source** | **GenBank**  **accession no.** |
| --- | --- | --- | --- | --- | --- | --- | --- | --- | --- |
| T0131 | Genotype 1 | CC8 | ST239 | MRSA | III | t030 | China | Human | CP002643.1 |
| TW20 | Genotype 1 | CC8 | ST239 | MRSA | III | t037 | UK | Human | FN433596.1 |
| JKD6008 | Genotype 1 | CC8 | ST239 | MRSA | III | t037 | New Zealand | Human | CP002120.1 |
| Z172 | Genotype 1 | CC8 | ST239 | MRSA | III | t037 | Taiwan | Human | CP006838.1 |
| XN108 | Genotype 1 | CC8 | ST239 | MRSA | III | t037 | China | Human | CP007447.1 |
| Gv69 | Genotype 1 | CC8 | ST239 | MRSA | III | t037 | Brazil | Human | CP009681.1 |
| Bmb9393 | Genotype 1 | CC8 | ST239 | MRSA | III | t138 | Brazil | Human | CP005288.1 |
| 04-02981 | Genotype 3 | CC5 | ST225 | MRSA | II | t003 | Germany | Human | CP001844.2 |
| 16035 | Genotype 3 | CC5 | ST228 | MRSA | I | t041 | Switzerland | Human | HE579065.1 |
| 16125 | Genotype 3 | CC5 | ST228 | MRSA | I | t041 | Switzerland | Human | HE579067.1 |
| 18583 | Genotype 3 | CC5 | ST228 | MRSA | I | t041 | Switzerland | Human | HE579073.1 |
| 18412 | Genotype 3 | CC5 | ST228 | MRSA | I | t041 | Switzerland | Human | HE579059.1 |
| 18341 | Genotype 3 | CC5 | ST228 | MRSA | I | t041 | Switzerland | Human | HE579061.1 |
| 15532 | Genotype 3 | CC5 | ST228 | MRSA | I | t041 | Switzerland | Human | HE579063.1 |
| 10497 | Genotype 3 | CC5 | ST228 | MRSA | I | t041 | Switzerland | Human | HE579069.1 |
| 10388 | Genotype 3 | CC5 | ST228 | MRSA | I | t041 | Switzerland | Human | HE579071.1 |
| N315 | Genotype 3 | CC5 | ST5 | MRSA | II | t002 | Japan | Human | BA000018.3 |
| Mu50 | Genotype 3 | CC5 | ST5 | MRSA | II | t002 | Japan | Human | BA000017.4 |
| Mu3 | Genotype 3 | CC5 | ST5 | MRSA | II | t002 | Japan | Human | AP009324.1 |
| JH9 | Genotype 3 | CC5 | ST5 | MRSA | II | t002 | USA | Human | CP000703.1 |
| JH1 | Genotype 3 | CC5 | ST5 | MRSA | II | t002 | USA | Human | CP000736.1 |
| ED98 | Genotype 3 | CC5 | ST5 | MRSA | II | t002 | Ireland | Animal | CP001781.1 |
| FCFHV36 | Genotype 3 | CC5 | ST105 | MRSA | II | t002 | Brazil | Human | CP011147.1 |
| CN1 | Genotype 3 | CC72 | ST72 | MRSA | IV | t324 | Korea | Human | CP003979.1 |
| 11819-97 | Genotype 3 | CC80 | ST80 | MRSA | IV | t044 | Denmark | Human | CP003194.1 |
| ECT-R 2 | Genotype 3 | CC5 | ST5 | MSSA | N/A | t002 | Sweden | Human | FR714927.1 |
| RKI4 | Genotype 3 | CC27 | ST27 | MSSA | N/A | t733 | Germany | Human | CP011528.1 |
| 502A | Genotype 3 | CC5 | ST5 | MSSA | N/A | t010 | USA | Human | CP007454.1 |
| SA40 | Genotype 4 | CC59 | ST59 | MRSA | IV | t441 | Taiwan | Human | CP003604.1 |
| SA268 | Genotype 4 | CC59 | ST59 | MRSA | IV | t441 | China | Human | CP006630.1 |
| M013 | Genotype 4 | CC59 | ST59 | MRSA | V | t437 | Taiwan | Human | CP003166.1 |
| SA957 | Genotype 4 | CC59 | ST59 | MRSA | V | t437 | Taiwan | Human | CP003603.1 |
| MW2 | Genotype 5 | CC1 | ST1 | MRSA | IV | t131 | USA | Human | BA000033.2 |
| MSSA476 | Genotype 5 | CC1 | ST1 | MSSA | N/A | t607 | UK | Human | BX571857.1 |
| COL | Genotype 7 | CC8 | ST250 | MRSA | I | t001 | UK | Environment | CP000046.1 |
| NRS 100 | Genotype 7 | CC8 | ST250 | MRSA | I | t008 | USA | NA | CP007539.1 |
| FPR3757 | Genotype 7 | CC8 | ST8 | MRSA | IV | t008 | USA | Human | CP000255.1 |
| ISMMS1 | Genotype 7 | CC8 | ST8 | MRSA | IV | t008 | USA | Human | CP007176.1 |
| 29b_MRSA | Genotype 7 | CC8 | ST8 | MRSA | IV | t008 | USA | Human | CP010295.1 |
| 31b_MRSA | Genotype 7 | CC8 | ST8 | MRSA | IV | t008 | USA | Human | CP010296.1 |
| 33b | Genotype 7 | CC8 | ST8 | MRSA | IV | t008 | USA | Human | CP010297.1 |
| 26b_MRSA | Genotype 7 | CC8 | ST8 | MRSA | IV | t008 | USA | Human | CP010298.1 |
| 25b_MRSA | Genotype 7 | CC8 | ST8 | MRSA | IV | t008 | USA | Human | CP010299.1 |
| 27b_MRSA | Genotype 7 | CC8 | ST8 | MRSA | IV | t008 | USA | Human | CP010300.1 |
| 2395 | Genotype 7 | CC8 | ST8 | MRSA | IV | t064 | USA | Human | CP007499.1 |
| TCH1516 | Genotype 7 | CC8 | ST8 | MRSA | IV | t622 | USA | Human | CP000730.1 |
| UA-S391 | Genotype 7 | CC80 | ST80 | MRSA | IV | t622 | Belgium | Human | CP007690.1 |
| M121 | Genotype 7 | CC8 | ST8 | MRSA | IV | t008 | NA | Human | CP007670.1 |
| CA15 | Genotype 7 | CC8 | ST8 | MRSA | IV | t008 | Colombia | Human | CP007674.1 |
| DSM 20231 | Genotype 7 | CC8 | ST8 | MSSA | N/A | t1029 | UK | Human | CP011526.1 |
| Newman | Genotype 7 | CC8 | ST8 | MSSA | N/A | t008 | UK | Human | AP009351.1 |
| NCTC 8325 | Genotype 7 | CC8 | ST8 | MSSA | N/A | t211 | UK | Human | CP000253.1 |
| VC40 | Genotype 7 | CC8 | ST8 | MSSA | N/A | t211 | Germany | Artificial | CP003033.1 |
| RF122 | Genotype 9 | CC705 | ST151 | MSSA | N/A | t529 | Ireland | Animal | AJ938182.1 |
| ED133 | Genotype 10 | CC133 | ST133 | MRSA | IV | t2678 | France | Animal | CP001996.1 |
| MSHR1132 | Genotype 11 | CC75 | ST1850 | MRSA | IV | NT | Australia | Human | FR821777.2 |
| ATCC 25923 | Genotype 12 | CC30 | ST243 | MSSA | N/A | t021 | USA | Human | CP009361.1 |
| MRSA252 | Genotype 13 | CC30 | ST36 | MRSA | II | t016 | UK | Human | BX571856.1 |
| FORC_001 | Genotype 14 | CC30 | ST30 | MSSA | N/A | t338 | South Korea | Plant | CP009554.1 |
| TCH60 | Genotype 14 | CC30 | ST30 | MRSA | IV | t019 | USA | Human | CP002110.1 |
| 55/2053 | Genotype 14 | CC30 | ST30 | MRSA | IV | t021 | NA | NA | CP002388.1 |
| CA-347 | Genotype 15 | CC45 | ST45 | MRSA | II | t004 | USA | Human | CP006044.1 |
| S0385 | Genotype 16 | CC398 | ST398 | MRSA | V | t011 | Netherlands | Human | AM990992.1 |
| 08BA02176 | Genotype 16 | CC398 | ST398 | MRSA | V | t034 | Canada | Human | CP003808.1 |
| LGA251 | Genotype 17 | CC425 | ST425 | MRSA | XI | t6300 | UK | Animal | FR821779.1 |
| DAR4145 | Genotype 18 | CC1 | ST772 | MRSA | V | t657 | India | Human | CP010526.1 |
| 6850 | Genotype 19 | CC50 | ST50 | MSSA | N/A | t185 | USA | Human | CP006706.1 |
| JKD6159 | Genotype 20 | CC93 | ST93 | MRSA | IV | t202 | Australia | Human | CP002114.2 |
| H-EMRSA-15 | Genotype 21 | CC22 | ST22 | MRSA | IV | NT | Belgium | Human | CP007659.1 |
| HO 5096 0412 | Genotype 21 | CC22 | ST22 | MRSA | IV | t1041 | UK | Human | HE681097.1 |

Abbreviations: N/A, not applicable; NA, data not available.
