# Supplementary material for: Genotypic Diversity of Staphylococcus aureus α-Hemolysin Gene (hla) and Its Association with Clonal Background: Implications for Vaccine Development
Source: PLoS One. 2016 Feb 11;11(2):e0149112. doi: 10.1371/journal.pone.0149112 (PMC4750931; doi:10.1371/journal.pone.0149112)
Supplement: S2 Table — (DOCX) [file pone.0149112.s002.docx]

**S2 Table. *In silico* analysis of *hla* genotype and clonal background of and 248 *S. aureus* genomes from four previous publications.**

| **Strain** | ***hla* genotype** | **Clonal complex**  **(CC)** | **Sequence type**  **(ST)** | **Phenotype** | ***spa* type** | **Country** | **Reference** |
| --- | --- | --- | --- | --- | --- | --- | --- |
| 09-00227 | Genotype 26 | CC121 | ST123 | MRSA | t171 | Germany | Kurt et al., 2013 |
| 09-02298 | Genotype 26 | CC121 | ST121 | MRSA | t159 | Tonga | Kurt et al., 2013 |
| 10-00259 | Genotype 26 | CC121 | ST121 | MRSA | t314 | Uganda | Kurt et al., 2013 |
| 10-00380 | Genotype 26 | CC121 | ST95 | MRSA | t645 | United Kingdom | Kurt et al., 2013 |
| 10-01133 | Genotype 26 | CC121 | ST121 | MRSA | t645 | Peru | Kurt et al., 2013 |
| 11-02371 | Genotype 26 | CC121 | ST121 | MRSA | t159 | Cambodia | Kurt et al., 2013 |
| 98-01618 | Genotype 26 | CC121 | ST121 | MRSA | t159 | Germany | Kurt et al., 2013 |
| TPS3104 | Genotype 20 | CC93 | ST93 | MRSA | t202 | Australia | Stinear et al., 2014 |
| TPS3105 | Genotype 20 | CC93 | ST93 | MRSA | t202 | Australia | Stinear et al., 2014 |
| TPS3106 | Genotype 20 | CC93 | ST93 | MRSA | t202 | Australia | Stinear et al., 2014 |
| TPS3132 | Genotype 20 | CC93 | ST93 | MRSA | t202 | Australia | Stinear et al., 2014 |
| TPS3133 | Genotype 20 | CC93 | ST93 | MRSA | t202 | Australia | Stinear et al., 2014 |
| TPS3134 | Genotype 20 | CC93 | ST93 | MRSA | t202 | Australia | Stinear et al., 2014 |
| TPS3135 | Genotype 20 | CC93 | ST93 | MRSA | t202 | Australia | Stinear et al., 2014 |
| TPS3136 | Genotype 20 | CC93 | ST93 | MRSA | t202 | Australia | Stinear et al., 2014 |
| TPS3137 | Genotype 20 | CC93 | ST93 | MRSA | t202 | Australia | Stinear et al., 2014 |
| TPS3138 | Genotype 20 | CC93 | ST93 | MRSA | t202 | Australia | Stinear et al., 2014 |
| TPS3139 | Genotype 20 | CC93 | ST93 | MRSA | t202 | Australia | Stinear et al., 2014 |
| TPS3140 | Genotype 20 | CC93 | ST93 | MRSA | t202 | Australia | Stinear et al., 2014 |
| TPS3142 | Genotype 20 | CC93 | ST93 | MRSA | t202 | Australia | Stinear et al., 2014 |
| TPS3144 | Genotype 20 | CC93 | ST93 | MRSA | t202 | Australia | Stinear et al., 2014 |
| TPS3145 | Genotype 20 | CC93 | ST93 | MRSA | t202 | Australia | Stinear et al., 2014 |
| TPS3146 | Genotype 20 | CC93 | ST93 | MRSA | t202 | Australia | Stinear et al., 2014 |
| TPS3147 | Genotype 20 | CC93 | ST93 | MRSA | t202 | Australia | Stinear et al., 2014 |
| TPS3148 | Genotype 20 | CC93 | ST93 | MRSA | t202 | Australia | Stinear et al., 2014 |
| TPS3149 | Genotype 20 | CC93 | ST93 | MRSA | t202 | Australia | Stinear et al., 2014 |
| TPS3150 | Genotype 20 | CC93 | ST93 | MRSA | t202 | Australia | Stinear et al., 2014 |
| TPS3151 | Genotype 20 | CC93 | ST93 | MRSA | t4178 | Australia | Stinear et al., 2014 |
| TPS3152 | Genotype 20 | CC93 | ST93 | MRSA | t202 | Australia | Stinear et al., 2014 |
| TPS3153 | Genotype 20 | CC93 | ST93 | MRSA | t202 | Australia | Stinear et al., 2014 |
| TPS3154 | Genotype 20 | CC93 | ST93 | MRSA | t202 | Australia | Stinear et al., 2014 |
| TPS3155 | Genotype 20 | CC93 | ST93 | MRSA | t202 | Australia | Stinear et al., 2014 |
| TPS3156 | Genotype 20 | CC93 | ST93 | MRSA | t202 | Australia | Stinear et al., 2014 |
| TPS3157 | Genotype 20 | CC93 | ST93 | MRSA | t202 | Australia | Stinear et al., 2014 |
| TPS3158 | Genotype 20 | CC93 | ST93 | MRSA | t202 | Australia | Stinear et al., 2014 |
| TPS3159 | Genotype 20 | CC93 | ST93 | MRSA | t202 | Australia | Stinear et al., 2014 |
| TPS3160 | Genotype 20 | CC93 | ST93 | MRSA | t202 | Australia | Stinear et al., 2014 |
| TPS3161 | Genotype 20 | CC93 | ST93 | MRSA | t1811 | Australia | Stinear et al., 2014 |
| TPS3162 | Genotype 20 | CC93 | ST93 | MRSA | t202 | Australia | Stinear et al., 2014 |
| TPS3163 | Genotype 20 | CC93 | ST93 | MRSA | t202 | Australia | Stinear et al., 2014 |
| TPS3164 | Genotype 20 | CC93 | ST93 | MRSA | t202 | Australia | Stinear et al., 2014 |
| TPS3165 | Genotype 20 | CC93 | ST93 | MRSA | t6487 | Australia | Stinear et al., 2014 |
| TPS3166 | Genotype 20 | CC93 | ST93 | MRSA | t202 | Australia | Stinear et al., 2014 |
| TPS3167 | Genotype 20 | CC93 | ST93 | MRSA | t202 | Australia | Stinear et al., 2014 |
| TPS3168 | Genotype 20 | CC93 | ST93 | MRSA | t202 | Australia | Stinear et al., 2014 |
| TPS3169 | Genotype 20 | CC93 | ST93 | MRSA | t202 | Australia | Stinear et al., 2014 |
| TPS3171 | Genotype 20 | CC93 | ST93 | MRSA | t202 | Australia | Stinear et al., 2014 |
| TPS3173 | Genotype 20 | CC93 | ST93 | MRSA | t202 | Australia | Stinear et al., 2014 |
| TPS3174 | Genotype 20 | CC93 | ST93 | MRSA | t202 | Australia | Stinear et al., 2014 |
| TPS3176 | Genotype 20 | CC93 | ST93 | MRSA | t202 | Australia | Stinear et al., 2014 |
| TPS3177 | Genotype 20 | CC93 | ST93 | MRSA | t202 | Australia | Stinear et al., 2014 |
| TPS3178 | Genotype 20 | CC93 | ST93 | MRSA | t5485 | Australia | Stinear et al., 2014 |
| TPS3179 | Genotype 20 | CC93 | ST93 | MRSA | t202 | Australia | Stinear et al., 2014 |
| TPS3181 | Genotype 20 | CC93 | ST93 | MRSA | t5767 | Australia | Stinear et al., 2014 |
| TPS3182 | Genotype 20 | CC93 | ST93 | MRSA | t202 | Australia | Stinear et al., 2014 |
| TPS3183 | Genotype 20 | CC93 | ST93 | MRSA | t202 | Australia | Stinear et al., 2014 |
| TPS3184 | Genotype 20 | CC93 | ST93 | MRSA | t202 | Australia | Stinear et al., 2014 |
| TPS3185 | Genotype 20 | CC93 | ST93 | MRSA | t5767 | Australia | Stinear et al., 2014 |
| TPS3186 | Genotype 20 | CC93 | ST93 | MRSA | t202 | Australia | Stinear et al., 2014 |
| TPS3187 | Genotype 20 | CC93 | ST93 | MRSA | t202 | Australia | Stinear et al., 2014 |
| TPS3188 | Genotype 20 | CC93 | ST93 | MRSA | t4178 | Australia | Stinear et al., 2014 |
| TPS3189 | Genotype 20 | CC93 | ST93 | MRSA | t4699 | Australia | Stinear et al., 2014 |
| 75916 | Genotype 13 | CC30 | ST36 | MRSA | NG | Finland | McAdam et al., 2012 |
| 01.7997.S | Genotype 14 | CC30 | ST30 | MRSA | NG | Scotland | McAdam et al., 2012 |
| 1227_07.08 | Genotype 13 | CC30 | ST36 | MRSA | NG | Scotland | McAdam et al., 2012 |
| 1696_07.08 | Genotype 13 | CC30 | ST36 | MRSA | NG | Scotland | McAdam et al., 2012 |
| 1712_07.08 | Genotype 13 | CC30 | ST36 | MRSA | NG | Scotland | McAdam et al., 2012 |
| 1791_03.08 | Genotype 13 | CC30 | ST36 | MRSA | NG | Scotland | McAdam et al., 2012 |
| 1973_07.17 | Genotype 14 | CC30 | ST30 | MRSA | NG | Scotland | McAdam et al., 2012 |
| 2449_07.17 | Genotype 13 | CC30 | ST36 | MRSA | NG | Scotland | McAdam et al., 2012 |
| 2496_07.17 | Genotype 13 | CC30 | ST36 | MRSA | NG | Scotland | McAdam et al., 2012 |
| 2589_07.17 | Genotype 13 | CC30 | ST36 | MRSA | NG | Scotland | McAdam et al., 2012 |
| 2880_07.25 | Genotype 13 | CC30 | ST36 | MRSA | NG | Scotland | McAdam et al., 2012 |
| 3366_99 | Genotype 13 | CC30 | ST36 | MRSA | NG | Ireland | McAdam et al., 2012 |
| 3841_07.33 | Genotype 13 | CC30 | ST36 | MRSA | NG | Scotland | McAdam et al., 2012 |
| 55_2053 | Genotype 14 | CC30 | ST30 | MRSA | NG | England | McAdam et al., 2012 |
| 5806_98.83 | Genotype 13 | CC30 | ST36 | MRSA | NG | Scotland | McAdam et al., 2012 |
| 58-362 | Genotype 14 | CC30 | ST30 | MRSA | NG | USA | McAdam et al., 2012 |
| 58-424 | Genotype 14 | CC30 | ST30 | MRSA | NG | USA | McAdam et al., 2012 |
| 65-1322 | Genotype 14 | CC30 | ST30 | MRSA | NG | USA | McAdam et al., 2012 |
| 65-20 | Genotype 14 | CC30 | ST30 | MRSA | NG | USA | McAdam et al., 2012 |
| 66-1888 | Genotype 14 | CC30 | ST30 | MRSA | NG | USA | McAdam et al., 2012 |
| 6636_07.67 | Genotype 13 | CC30 | ST36 | MRSA | NG | Scotland | McAdam et al., 2012 |
| 6659_07.67 | Genotype 13 | CC30 | ST36 | MRSA | NG | Scotland | McAdam et al., 2012 |
| 68-397 | Genotype 14 | CC30 | ST30 | MRSA | NG | USA | McAdam et al., 2012 |
| 69-172 | Genotype 14 | CC30 | ST30 | MRSA | NG | USA | McAdam et al., 2012 |
| 69-412 | Genotype 14 | CC30 | ST30 | MRSA | NG | USA | McAdam et al., 2012 |
| 7206_07.75 | Genotype 13 | CC30 | ST36 | MRSA | NG | Scotland | McAdam et al., 2012 |
| 7G_02 | Genotype 13 | CC30 | ST500 | MRSA | NG | USA | McAdam et al., 2012 |
| 9570_06.92 | Genotype 13 | CC30 | ST36 | MRSA | NG | Scotland | McAdam et al., 2012 |
| A017934_97 | Genotype 14 | CC30 | ST30 | MRSA | NG | Sweden | McAdam et al., 2012 |
| DEN4415 | Genotype 13 | CC30 | ST36 | MRSA | NG | Denmark | McAdam et al., 2012 |
| E1410 | Genotype 14 | CC30 | ST30 | MRSA | NG | Denmark | McAdam et al., 2012 |
| HAR24 | Genotype 13 | CC30 | ST36 | MRSA | NG | UK | McAdam et al., 2012 |
| HU275 | Genotype 13 | CC30 | ST36 | MRSA | NG | Hungary | McAdam et al., 2012 |
| M1015 | Genotype 14 | CC30 | ST30 | MRSA | NG | Australia | McAdam et al., 2012 |
| M1016 | Genotype 14 | CC30 | ST30 | MRSA | NG | Australia | McAdam et al., 2012 |
| M876 | Genotype 14 | CC30 | ST30 | MRSA | NG | Australia | McAdam et al., 2012 |
| M899 | Genotype 14 | CC30 | ST30 | MRSA | NG | Australia | McAdam et al., 2012 |
| WBG10049 | Genotype 14 | CC30 | ST30 | MRSA | NG | Australia | McAdam et al., 2012 |
| T12N1 | Genotype 1 | CC8 | ST239 | MRSA | NG | Thailand | Tong et al., 2015 |
| T12N24 | Genotype 1 | CC8 | ST239 | MRSA | NG | Thailand | Tong et al., 2015 |
| T12N28 | Genotype 1 | CC8 | ST239 | MRSA | NG | Thailand | Tong et al., 2015 |
| T178N3 | Genotype 1 | CC8 | ST239 | MRSA | NG | Thailand | Tong et al., 2015 |
| T182N1 | Genotype 1 | CC8 | ST239 | MRSA | NG | Thailand | Tong et al., 2015 |
| T183N2 | Genotype 1 | CC8 | ST239 | MRSA | NG | Thailand | Tong et al., 2015 |
| T183N8 | Genotype 1 | CC8 | ST239 | MRSA | NG | Thailand | Tong et al., 2015 |
| T188N1 | Genotype 1 | CC8 | ST239 | MRSA | NG | Thailand | Tong et al., 2015 |
| T188N3 | Genotype 1 | CC8 | ST239 | MRSA | NG | Thailand | Tong et al., 2015 |
| T192T3 | Genotype 1 | CC8 | ST239 | MRSA | NG | Thailand | Tong et al., 2015 |
| T194N1 | Genotype 1 | CC8 | ST239 | MRSA | NG | Thailand | Tong et al., 2015 |
| T194N4 | Genotype 1 | CC8 | ST239 | MRSA | NG | Thailand | Tong et al., 2015 |
| T197N1 | Genotype 1 | CC8 | ST239 | MRSA | NG | Thailand | Tong et al., 2015 |
| T197N3 | Genotype 1 | CC8 | ST239 | MRSA | NG | Thailand | Tong et al., 2015 |
| T20N1 | Genotype 1 | CC8 | ST239 | MRSA | NG | Thailand | Tong et al., 2015 |
| T20N2 | Genotype 1 | CC8 | ST239 | MRSA | NG | Thailand | Tong et al., 2015 |
| T223A1 | Genotype 1 | CC8 | ST239 | MRSA | NG | Thailand | Tong et al., 2015 |
| T225N1 | Genotype 1 | CC8 | ST239 | MRSA | NG | Thailand | Tong et al., 2015 |
| T232N2 | Genotype 1 | CC8 | ST239 | MRSA | NG | Thailand | Tong et al., 2015 |
| T232T1 | Genotype 1 | CC8 | ST239 | MRSA | NG | Thailand | Tong et al., 2015 |
| T234N4 | Genotype 1 | CC8 | ST239 | MRSA | NG | Thailand | Tong et al., 2015 |
| T249N3 | Genotype 1 | CC8 | ST239 | MRSA | NG | Thailand | Tong et al., 2015 |
| T249T1 | Genotype 1 | CC8 | ST239 | MRSA | NG | Thailand | Tong et al., 2015 |
| T270U1 | Genotype 1 | CC8 | ST239 | MRSA | NG | Thailand | Tong et al., 2015 |
| T271C1 | Genotype 1 | CC8 | ST239 | MRSA | NG | Thailand | Tong et al., 2015 |
| T271N4 | Genotype 1 | CC8 | ST239 | MRSA | NG | Thailand | Tong et al., 2015 |
| T28C2 | Genotype 1 | CC8 | ST239 | MRSA | NG | Thailand | Tong et al., 2015 |
| T301T2 | Genotype 1 | CC8 | ST239 | MRSA | NG | Thailand | Tong et al., 2015 |
| T303N3 | Genotype 1 | CC8 | ST239 | MRSA | NG | Thailand | Tong et al., 2015 |
| T322N2 | Genotype 1 | CC8 | ST239 | MRSA | NG | Thailand | Tong et al., 2015 |
| T327N2 | Genotype 1 | CC8 | ST239 | MRSA | NG | Thailand | Tong et al., 2015 |
| T330T1 | Genotype 1 | CC8 | ST239 | MRSA | NG | Thailand | Tong et al., 2015 |
| T335A2 | Genotype 1 | CC8 | ST239 | MRSA | NG | Thailand | Tong et al., 2015 |
| T358N1 | Genotype 1 | CC8 | ST239 | MRSA | NG | Thailand | Tong et al., 2015 |
| T358N2 | Genotype 1 | CC8 | ST239 | MRSA | NG | Thailand | Tong et al., 2015 |
| T35N2 | Genotype 1 | CC8 | ST239 | MRSA | NG | Thailand | Tong et al., 2015 |
| T65N2 | Genotype 1 | CC8 | ST239 | MRSA | NG | Thailand | Tong et al., 2015 |
| T65N3 | Genotype 1 | CC8 | ST239 | MRSA | NG | Thailand | Tong et al., 2015 |
| T69N1 | Genotype 1 | CC8 | ST239 | MRSA | NG | Thailand | Tong et al., 2015 |
| T71N2 | Genotype 1 | CC8 | ST239 | MRSA | NG | Thailand | Tong et al., 2015 |
| T71N3 | Genotype 1 | CC8 | ST239 | MRSA | NG | Thailand | Tong et al., 2015 |
| T95N2 | Genotype 1 | CC8 | ST239 | MRSA | NG | Thailand | Tong et al., 2015 |
| T99N5 | Genotype 1 | CC8 | ST239 | MRSA | NG | Thailand | Tong et al., 2015 |
| 03-00220 | Genotype 24 | CC22 | ST22 | MSSA | t005 | Germany | Holden et al., 2013 |
| 03-00397 | Genotype 25 | CC22 | ST22 | MRSA | t032 | Germany | Holden et al., 2013 |
| 03-01478 | Genotype 24 | CC22 | ST22 | MRSA | t032 | Germany | Holden et al., 2013 |
| 04-00608 | Genotype 24 | CC22 | ST22 | MRSA | t310 | Germany | Holden et al., 2013 |
| 04-00609 | Genotype 22 | CC22 | ST22 | MRSA | t309 | Germany | Holden et al., 2013 |
| 04-02314-1 | Genotype 22 | CC22 | ST22 | MRSA | t849 | UK | Holden et al., 2013 |
| 04-02315-1 | Genotype 27 | CC22 | ST22 | MRSA | t005 | UK | Holden et al., 2013 |
| 04-03100 | Genotype 22 | CC22 | ST22 | MRSA | t032 | Germany | Holden et al., 2013 |
| 04-03103 | Genotype 28 | CC22 | ST22 | MRSA | t032 | Germany | Holden et al., 2013 |
| 04-03111 | Genotype 24 | CC22 | ST22 | MRSA | t032 | Germany | Holden et al., 2013 |
| 05-01089 | Genotype 24 | CC22 | ST22 | MRSA | t310 | Germany | Holden et al., 2013 |
| 06-00896 | Genotype 21 | CC22 | ST22 | MRSA | t310 | Germany | Holden et al., 2013 |
| 06-01347 | Genotype 25 | CC22 | ST22 | MSSA | t712 | Germany | Holden et al., 2013 |
| 06-01396 | Genotype 27 | CC22 | ST22 | MSSA | t1021 | Germany | Holden et al., 2013 |
| 06-01650 | Genotype 22 | CC22 | ST22 | MRSA | t022 | Germany | Holden et al., 2013 |
| 06-01686 | Genotype 24 | CC22 | ST22 | MRSA | t476 | Germany | Holden et al., 2013 |
| 06-01900 | Genotype 24 | CC22 | ST22 | MRSA | t032 | Germany | Holden et al., 2013 |
| 06-02400 | Genotype 25 | CC22 | ST22 | MRSA | t1214 | Germany | Holden et al., 2013 |
| 06-02459 | Genotype 24 | CC22 | ST22 | MRSA | t025 | Germany | Holden et al., 2013 |
| 06-02751 | Genotype 24 | CC22 | ST22 | MRSA | t025 | Germany | Holden et al., 2013 |
| 07-00058 | Genotype 22 | CC22 | ST22 | MRSA | t032 | Germany | Holden et al., 2013 |
| 07-00059 | Genotype 25 | CC22 | ST22 | MRSA | t032 | Germany | Holden et al., 2013 |
| 07-00060 | Genotype 23 | CC22 | ST22 | MRSA | t032 | Germany | Holden et al., 2013 |
| 07-00061 | Genotype 21 | CC22 | ST22 | MRSA | t032 | Germany | Holden et al., 2013 |
| 07-00062 | Genotype 22 | CC22 | ST22 | MRSA | t032 | Germany | Holden et al., 2013 |
| 07-00063 | Genotype 25 | CC22 | ST22 | MRSA | t032 | Germany | Holden et al., 2013 |
| 07-00064 | Genotype 23 | CC22 | ST22 | MRSA | t032 | Germany | Holden et al., 2013 |
| 07-00309 | Genotype 22 | CC22 | ST22 | MRSA | t032 | Germany | Holden et al., 2013 |
| 07-00655 | Genotype 25 | CC22 | ST22 | MRSA | t025 | Germany | Holden et al., 2013 |
| 07-01319 | Genotype 24 | CC22 | ST22 | MRSA | t025 | Germany | Holden et al., 2013 |
| 07-01497 | Genotype 28 | CC22 | ST22 | MRSA | t032 | Germany | Holden et al., 2013 |
| 07-01744 | Genotype 24 | CC22 | ST22 | MRSA | t432 | Nigeria | Holden et al., 2013 |
| 07-01932 | Genotype 24 | CC22 | ST22 | MSSA | t223 | Sudan | Holden et al., 2013 |
| 07-02088 | Genotype 21 | CC22 | ST22 | MRSA | t025 | Germany | Holden et al., 2013 |
| 07-02159 | Genotype 21 | CC22 | ST22 | MRSA | t005 | Germany | Holden et al., 2013 |
| 07-02389 | Genotype 25 | CC22 | ST22 | MSSA | t005 | Germany | Holden et al., 2013 |
| 07-02477 | Genotype 23 | CC22 | ST22 | MRSA | t032 | Germany | Holden et al., 2013 |
| 07-02789 | Genotype 24 | CC22 | ST22 | MRSA | t025 | Germany | Holden et al., 2013 |
| 07-02901 | Genotype 24 | CC22 | ST22 | MRSA | t310 | Germany | Holden et al., 2013 |
| 07-02986 | Genotype 24 | CC22 | ST22 | MRSA | t032 | Australia | Holden et al., 2013 |
| 07-02987 | Genotype 24 | CC22 | ST22 | MRSA | t1963 | Australia | Holden et al., 2013 |
| 07-02988 | Genotype 21 | CC22 | ST22 | MRSA | t032 | Australia | Holden et al., 2013 |
| 07-02989 | Genotype 21 | CC22 | ST22 | MRSA | t032 | Australia | Holden et al., 2013 |
| 07-02990 | Genotype 24 | CC22 | ST22 | MRSA | t032 | Australia | Holden et al., 2013 |
| 07-02992 | Genotype 24 | CC22 | ST22 | MRSA | t032 | Australia | Holden et al., 2013 |
| 07-02994 | Genotype 24 | CC22 | ST22 | MRSA | t032 | Australia | Holden et al., 2013 |
| 07-02995 | Genotype 24 | CC22 | ST22 | MRSA | t032 | Australia | Holden et al., 2013 |
| 07-02996 | Genotype 22 | CC22 | ST22 | MRSA | t3211 | Australia | Holden et al., 2013 |
| 07-02997 | Genotype 25 | CC22 | ST22 | MRSA | t3212 | Australia | Holden et al., 2013 |
| 07-03135 | Genotype 21 | CC22 | ST22 | MSSA | t005 | Germany | Holden et al., 2013 |
| 07-03339 | Genotype 21 | CC22 | ST22 | MRSA | t032 | Czech Republic | Holden et al., 2013 |
| 07-03341 | Genotype 24 | CC22 | ST22 | MRSA | t032 | Czech Republic | Holden et al., 2013 |
| 07-03345 | Genotype 24 | CC22 | ST22 | MRSA | t032 | Czech Republic | Holden et al., 2013 |
| 07-03346 | Genotype 24 | CC22 | ST22 | MRSA | t032 | Czech Republic | Holden et al., 2013 |
| 07-03349 | Genotype 21 | CC22 | ST22 | MRSA | t032 | Denmark | Holden et al., 2013 |
| 07-03351 | Genotype 27 | CC22 | ST22 | MRSA | t022 | Denmark | Holden et al., 2013 |
| 07-03353 | Genotype 22 | CC22 | ST22 | MRSA | t032 | Denmark | Holden et al., 2013 |
| 07-03354-2 | Genotype 24 | CC22 | ST22 | MRSA | t005 | Denmark | Holden et al., 2013 |
| 07-03356 | Genotype 25 | CC22 | ST22 | MSSA | t005 | Denmark | Holden et al., 2013 |
| 08-00602 | Genotype 25 | CC22 | ST22 | MRSA | t025 | Germany | Holden et al., 2013 |
| 08-01304 | Genotype 25 | CC22 | ST22 | MRSA | t025 | Germany | Holden et al., 2013 |
| 08-01482 | Genotype 25 | CC22 | ST22 | MRSA | t032 | Portugal | Holden et al., 2013 |
| 08-01484 | Genotype 22 | CC22 | ST22 | MRSA | t032 | Portugal | Holden et al., 2013 |
| 08-01485 | Genotype 24 | CC22 | ST22 | MRSA | t747 | Portugal | Holden et al., 2013 |
| 08-01486 | Genotype 25 | CC22 | ST22 | MRSA | t032 | Portugal | Holden et al., 2013 |
| 08-01488 | Genotype 24 | CC22 | ST22 | MRSA | t032 | Portugal | Holden et al., 2013 |
| 08-01489 | Genotype 22 | CC22 | ST22 | MRSA | t032 | Hungary | Holden et al., 2013 |
| 08-01490 | Genotype 25 | CC22 | ST22 | MSSA | t790 | Portugal | Holden et al., 2013 |
| 08-01491 | Genotype 25 | CC22 | ST22 | MRSA | t032 | Portugal | Holden et al., 2013 |
| 08-01492 | Genotype 21 | CC22 | ST22 | MSSA | t852 | Portugal | Holden et al., 2013 |
| 08-01666 | Genotype 25 | CC22 | ST22 | MRSA | t1214 | Germany | Holden et al., 2013 |
| 08-01667 | Genotype 25 | CC22 | ST22 | MRSA | t1214 | Germany | Holden et al., 2013 |
| 08-01668 | Genotype 25 | CC22 | ST22 | MRSA | t032 | Germany | Holden et al., 2013 |
| 08-01670 | Genotype 23 | CC22 | ST22 | MRSA | t032 | Germany | Holden et al., 2013 |
| 08-01671 | Genotype 24 | CC22 | ST22 | MRSA | t032 | Germany | Holden et al., 2013 |
| 08-01673 | Genotype 21 | CC22 | ST22 | MRSA | t531 | Germany | Holden et al., 2013 |
| 08-01674 | Genotype 25 | CC22 | ST22 | MRSA | t432 | Germany | Holden et al., 2013 |
| 09-00678 | Genotype 21 | CC22 | ST22 | MSSA | t891 | Namibia | Holden et al., 2013 |
| 09-00683 | Genotype 25 | CC22 | ST22 | MSSA | t005 | Namibia | Holden et al., 2013 |
| 09-00980 | Genotype 27 | CC22 | ST22 | MRSA | t022 | UK | Holden et al., 2013 |
| 09-00981 | Genotype 21 | CC22 | ST22 | MRSA | t032 | New Zealand | Holden et al., 2013 |
| 09-00983 | Genotype 21 | CC22 | ST22 | MRSA | t032 | New Zealand | Holden et al., 2013 |
| 09-00984 | Genotype 24 | CC22 | ST22 | MRSA | t032 | New Zealand | Holden et al., 2013 |
| 09-00985 | Genotype 25 | CC22 | ST22 | MSSA | t005 | Australia | Holden et al., 2013 |
| 09-01244 | Genotype 25 | CC22 | ST22 | MRSA | t032 | Singapore | Holden et al., 2013 |
| 98-10618 | Genotype 25 | CC22 | ST22 | MRSA | t022 | UK | Holden et al., 2013 |
| 98-26821 | Genotype 25 | CC22 | ST22 | MRSA | t1275 | UK | Holden et al., 2013 |
| 99St18131 | Genotype 24 | CC22 | ST22 | MRSA | t032 | Australia | Holden et al., 2013 |
| ARI1 | Genotype 21 | CC22 | ST22 | MRSA | t379 | UK | Holden et al., 2013 |
| ARI10 | Genotype 21 | CC22 | ST22 | MRSA | t032 | UK | Holden et al., 2013 |
| ARI11 | Genotype 21 | CC22 | ST22 | MRSA | t790 | UK | Holden et al., 2013 |
| ARI12 | Genotype 21 | CC22 | ST22 | MRSA | t022 | UK | Holden et al., 2013 |
| ARI26 | Genotype 21 | CC22 | ST22 | MRSA | t032 | UK | Holden et al., 2013 |
| ARI29 | Genotype 21 | CC22 | ST22 | MRSA | t032 | UK | Holden et al., 2013 |
| ARI31 | Genotype 21 | CC22 | ST22 | MRSA | t032 | UK | Holden et al., 2013 |
| ARI5 | Genotype 21 | CC22 | ST22 | MRSA | t032 | UK | Holden et al., 2013 |
| C720 | Genotype 21 | CC22 | ST22 | MRSA | t032 | UK | Holden et al., 2013 |
| F902 | Genotype 25 | CC22 | ST22 | MRSA | t032 | UK | Holden et al., 2013 |
| H65 | Genotype 21 | CC22 | ST22 | MSSA | t7741 | UK | Holden et al., 2013 |
| H914 | Genotype 22 | CC22 | ST22 | MRSA | t032 | UK | Holden et al., 2013 |
| M665 | Genotype 28 | CC22 | ST22 | MRSA | t032 | UK | Holden et al., 2013 |
| M810 | Genotype 21 | CC22 | ST22 | MRSA | t032 | UK | Holden et al., 2013 |
| NL | Genotype 21 | CC22 | ST22 | MSSA | t005 | Netherlands | Holden et al., 2013 |
| T505 | Genotype 23 | CC22 | ST22 | MRSA | t022 | UK | Holden et al., 2013 |
| W449 | Genotype 24 | CC22 | ST22 | MRSA | t022 | UK | Holden et al., 2013 |

Abbreviation: NG, not given.
